# 2-Methoxyoestradiol-3,17-*O*,*O*-*bis*-sulphamate and 2-deoxy-D-glucose in combination: a potential treatment for breast and prostate cancer

**DOI:** 10.1038/sj.bjc.6604752

**Published:** 2008-11-04

**Authors:** S L C Tagg, P A Foster, M P Leese, B V L Potter, M J Reed, A Purohit, S P Newman

**Affiliations:** 1 Oncology Drug Discovery and Women's Health Group, Faculty of Medicine, Imperial College London, St Mary's Hospital, London W2 1NY, UK; 2 Medicinal Chemistry and Sterix Ltd, Department of Pharmacy and Pharmacology, University of Bath, Claverton Down, Bath BA2 7AY, UK

**Keywords:** 2-methoxyoestradiol-3,17-*O*,*O*-*bis*-sulphamate, 2-deoxy-D-glucose, breast, prostate, microtubule

## Abstract

Drug combination therapy is a key strategy to improve treatment efficacy and survival of cancer patients. In this study the effects of combining 2-methoxyoestradiol-3,17-*O*,*O*-*bis*-sulphamate (STX140), a microtubule disruptor, with 2-deoxy-D-glucose (2DG) were assessed in MCF-7 (breast) and LNCaP (prostate) xenograft models *in vivo*. In mice bearing MCF-7 xenografts, daily p.o. administration of STX140 (5 mg kg^−1^) resulted in a 46% (*P*<0.05) reduction of tumour volume. However, the combination of STX140 (5 mg kg^−1^ p.o.) and 2DG (2 g kg^−1^ i.p.) reduced tumour volume by 76% (*P*<0.001). 2-Methoxyoestradiol-3,17-*O*,*O*-*bis*-sulphamate also reduced tumour vessel density. 2-Deoxy-D-glucose alone had no significant effect on tumour volume or vessel density. A similar benefit of the combination treatment was observed in the LNCaP prostate xenograft model. *In vitro* the degree of inhibition of cell proliferation by STX140 was unaffected by oxygen concentrations. In contrast, the inhibition of proliferation by 2DG was enhanced under hypoxia by 20 and 25% in MCF-7 and LNCaP cells, respectively. The combination of STX140 and 2DG in LNCaP cells under normoxia or hypoxia inhibited proliferation to a greater extent than either compound alone. These results suggest that the antiangiogenic and microtubule disruption activities of STX140 may make tumours more susceptible to inhibition of glycolysis by 2DG. This is the first study to show the benefit of combining a microtubule disruptor with 2DG in the two most common solid tumours.

A majority of both breast and prostate tumours initially present as hormone-dependent diseases and hormone deprivation therapy is efficacious. However, a significant number of these tumours will become hormone-independent and require more aggressive treatments. Current treatments for advanced breast and prostate cancer are limited, with resistance and toxicity being common problems with many therapies (Gottesman *et al*, 2002). Solid tumours are particularly difficult to treat because conventional chemotherapeutic drugs and radiation therapy only target the rapidly growing peripheral cells, leaving the slower proliferating tumour cells in the hypoxic core to survive (Brown and Giaccia, 1998).

Normal healthy cells undergo aerobic respiration to produce ATP; however, Warburg showed that, even in the presence of oxygen, cancer cells create ATP anaerobically, relying on glycolysis, the Warburg effect (Warburg and Dickens, 1930). Hence, inhibiting glycolysis could specifically target cancer cells without having deleterious effects on normal cells. Glycolysis is inhibited by using the glucose analogue, 2-deoxy-D-glucose (2DG), where the C-2 hydroxy group of glucose is replaced by a hydrogen (Figure 1, A and B). Both glucose and 2DG are phosphorylated by hexokinase; however, in contrast to glucose 6-phosphate, 2DG 6-phosphate cannot be further metabolised to fructose-6-phosphate by phosphoglucose isomerase (Nirenberg and Hogg, 1958). In addition to blocking the glycolytic pathway, 2DG competes with glucose for uptake by the hexose (GLUT) transporters (Wachsberger *et al*, 2002), thus limiting the uptake of glucose by the cell.

Several studies have shown the antitumour activity of 2DG alone *in vitro* and *in vivo* (Kaplan *et al*, 1990; Liu *et al*, 2001; Aft *et al*, 2002; Maschek *et al*, 2004; Gupta *et al*, 2005; Lampidis *et al*, 2006). However, very few clinical studies have tested 2DG as a single-agent therapy and the results of such studies have been disappointing (Landau *et al*, 1958; Kaplan *et al*, 1990). Combining 2DG with other anticancer modalities, such as chemotherapy or radiotherapy, could improve the efficacy of anti-glycolysis-based therapy (Singh *et al*, 2005; Simons *et al*, 2007). Maschek *et al* (2004) hypothesised that chemotherapy may kill the rapidly growing cells as 2DG targets the slowly proliferating inner core of cells, which are more reliant on glycolysis. This hypothesis was supported by the observation that a combination of 2DG and the anthracycline, adriamycin, was significantly more efficacious than either agent alone in an osteosarcoma xenograft model. Preliminary studies by the same group, in a NSCLC xenograft model, also indicated a possible benefit of combining paclitaxel and 2DG (Maschek *et al*, 2004).

2-Methoxyoestradiol-3,17-*O*,*O*-*bis*-sulphamate (STX140) is an orally bioavailable microtubule disruptor that can be dosed daily *in vivo*, with little blood toxicity being observed (Ireson *et al*, 2004; Newman *et al*, 2007; Foster *et al*, 2008c). Microtubule disruption by STX140 leads to cell cycle arrest and subsequent apoptosis of both tumour and endothelial cells (Foster *et al*, 2008a). The proliferation of both breast, MCF-7 (ER-positive), cells (Newman *et al*, 2004) and prostate, LNCaP (AR-positive), cells (Day *et al*, 2003) is inhibited *in vitro* by STX140. Furthermore, STX140 inhibits both *in vitro* angiogenesis (Newman *et al*, 2004) and *in vivo* angiogenesis (Chander *et al*, 2007).

A potential strategy for treating solid tumours would be to combine an antiangiogenic–microtubule disruptor, STX140, with an inhibitor of glycolysis, 2DG. The inhibition of angiogenesis by STX140 may increase hypoxia and thus make the tumour even more reliant on glycolysis, in theory sensitising the tumour further to 2DG.

The efficacy of combining 2DG with a new-generation microtubule disruptor, STX140, in xenograft models of the most common solid tumours in women and men is reported for the first time in this study.

## Materials and methods

### Compounds

**Figure 1:**
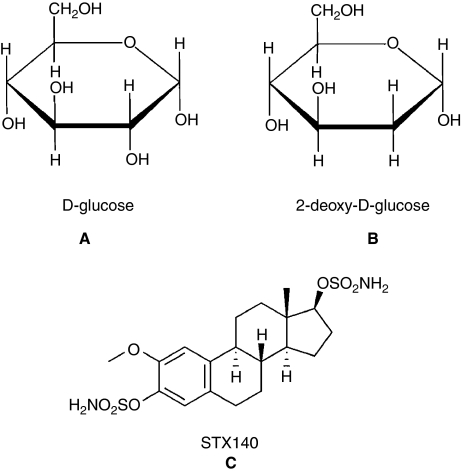
Structures of glucose (**A**) and 2DG (**B**). Glucose and 2DG differ at the second carbon. Chemical structure of STX140 (**C**).

2-Deoxy-D-glucose (Figure 1, B) was obtained from Sigma (Poole, UK). 2-Methoxyoestradiol-3,17-*O*,*O*-*bis*-sulphamate (Figure 1, C) was synthesised as described previously (Leese *et al*, 2006). 2-Methoxyoestradiol-3,17-*O*,*O*-*bis*-sulphamate yielded spectroscopic and analytical data in accordance with its structure.

### Cell culture

MCF-7 and LNCaP cells were obtained from the American Tissue Culture Collection (LGC Promochem, Teddington, UK). Cells were cultured in the following medium obtained from Sigma: RPMI 1640 with 10% foetal bovine serum, 1% L-glutamine, 1% MEM non-essential amino acids and 1% sodium bicarbonate solution. LNCaP cells also required 1% sodium pyruvate. Cells were maintained in a humidified incubator at 37°C under 5% CO_2_. For hypoxic experiments an Innova CO-48 CO_2_ humidified incubator (Newbrunswick Scientific, St Albans, UK) was used, oxygen was maintained at 1% and CO_2_ at 5% in a humidified atmosphere at 37°C.

### Proliferation assay

MCF-7 and LNCaP cells were seeded at 3000 and 5000 cells per well, respectively, in 96-well plates. To ensure that cells had adhered to the plate, compounds were not added until 4 h after the seeding of the cells. After 72–96 h cell numbers were measured by the addition of 10 *μ*l of Alamar blue (Biosource, Nivelles, Belgium) for 2 h and, subsequently, fluorescence was quantified using a FluoStar Optima (BMG Labtech, Offenburg, Germany) Plate reader (544 nm excitation; 590 nm emission).

### Measurement of ATP

MCF-7 and LNCaP cells were seeded at 3000 and 5000 cells per well, respectively, in 96-well plates. Compounds were added 4 h after seeding of cells. The ATPlite assay procedure was carried out according to the manufacturer's instructions (Perkin-Elmer, Beaconsfield, UK). The luminescence emitted from the ATP-dependent luciferase reaction was measured using a FluoStar Optima Plate reader.

### Cell cycle/apoptosis analysis

Cells were plated in T-25 flasks so that after 24 h they were 60–70% confluent and were then treated with the compounds for a further 48 h. Control cells were untreated or treated with tetrahydrofuran (THF) vehicle only. To harvest cells for flow cytometric DNA analysis, cells were washed with PBS before being trypsinised (0.25% trypsin, 0.05% EDTA). The medium containing non-adherent cells was also collected and pooled with the trypsinised cells. The cells and PBS-washings were pelleted by centrifugation at 1500 rpm and washed twice with PBS.

For cell cycle analysis the cells were then fixed in cold 70% ethanol, treated with 100 *μ*g ml^−1^ RNase for 5 min, stained with 50 *μ*g ml^−1^ propidium iodide and analysed using a flow cytometer (FACScan, Becton Dickinson, Cowley, UK).

For quantification of apoptosis the cells were re-suspended in binding buffer (10 mM HEPES/NaOH pH 7.4, 140 mM NaCl, 2.5 mM CaCl_2_) at 1 × 10^6^ cells ml^−1^. Cells were then stained with fluorescein-conjugated Annexin V (BD Biosciences, Cowley, UK) antibody and propidium iodide (5 *μ*g ml^−1^) before flow cytometric analysis. Apoptotic cells are defined as cells positive for Annexin V and negative for propidium iodide.

### *In vivo* tumour xenograft model

Intact, athymic, female and male MF-1 nude mice (nu−/nu−) were purchased from Harlan (Bicester, Oxon, UK) at 5 weeks of age (∼20–25 g in weight). All efforts were made to minimise both suffering and the number of animals used. Experiments were carried out under the UK Animals (Scientific Procedures) Act 1986 and complied with institutional guidelines. Animals were kept in a 12 h light/12 h dark cycle and given food and water *ad libitum*. Five million cells (MCF-7 or LNCaP), in ice-cold Matrigel (BD Biosciences), were inoculated s.c. into the right flank of the animals. Once the tumours reached 100–150 mm^3^ in volume, mice were randomly divided into four treatment groups (*n*=5):

1. Vehicle (10% THF; 90% propylene glycol (PG)), 100 *μ*l p.o. (oral gavage) + saline i.p. 200 *μ*l.
2. Vehicle p.o. + 2DG (2 g kg^−1^) i.p. 200 *μ*l.
3. STX140 (5 mg kg^−1^) 100 *μ*l p.o. + saline i.p. 200 *μ*l.
4. STX140 (5 mg kg^−1^) p.o. + 2DG (2 g kg^−1^) i.p. 200 *μ*l.

All treatments were administered daily for 4 weeks. Throughout the study mice were weighed and tumour measurements were taken on a weekly basis with electronic calipers. The dose of 2DG used was based on the work of Maschek *et al* (2004); STX140 was used at a quarter (5 mg kg^−1^ p.o.) of its optimal dose (20 mg kg^−1^ p.o.); hence, any additive effect with 2DG could easily be observed (Foster *et al*, 2008b). Tumour volumes were calculated using the formula length × width^2^/2. At the end of the study, MCF-7 tumours were placed in 10% formalin solution (Sigma) for immunohistochemical (IHC) analysis.

### Immunohistochemistry

von Willebrand's factor IHC analysis was performed on paraffin-embedded MCF-7 tumour sections cut at 6 *μ*m. After sectioning, rehydration and antigen retrieval steps, von Willebrand's antibody (1 : 800, Abcam, Cambridge, UK) was applied to the section for 1 h at RT, followed by a goat polyclonal secondary antibody conjugated to FITC (30 min at RT). In addition, sections were cut for routine H&E staining. Sections were viewed with a Zeiss Axiovert 200 fluorescence microscope (Carl Zeiss Ltd, Welwyn Garden City, UK) fitted with 20 × and 40 × Plan-Neofluor objectives. Images were captured using the Axiovision imaging system (Imaging Associates, Bicester, UK).

### Statistics

All experiments were carried out in triplicate and data presented are representative of one of three such experiments, unless indicated. All errors shown are the mean±s.e.m., unless otherwise stated. Student's *t-*test was used to assess the significance of the differences in cell proliferation, ATP levels *in vitro* and changes in tumour volume *in vivo.*

## Results

### Inhibition of proliferation by 2DG

**Figure 2:**
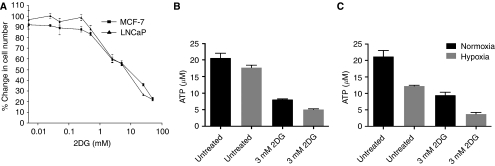
(**A**) Effect of 2DG on the proliferation of LNCaP and MCF-7 cells. Cells were cultured in 96-well plates and treated with 2DG (0.005–50 mM) for 96 h when their effects on proliferation were measured using a microtitre plate assay. Effect of 2DG and hypoxia on ATP levels in MCF-7 (**B**) and LNCaP cells (**C**). Cells were cultured in 96-well plates under normoxia and treated with 2DG (3 mM) for 72 h when their effects on ATP were measured using the microtitre, ATPlite plate assay. Results are expressed as percent of untreated. At this dose of 2DG, no significant inhibition of cell proliferation occurred (data not shown).

The ability of 2DG to inhibit the proliferation of MCF-7 and LNCaP cell lines was examined over a 96-h period under normoxic conditions (Figure 2A). The results show a reduction in MCF-7 and LNCaP cell proliferation with increasing 2DG concentration. The potency of 2DG was similar in both cell lines (MCF-7, IC_50_: 8.1 mM and LNCaP, IC_50_: 6.7 mM). The difference in IC_50_ values between MCF-7 and LNCaP cells was not significant. Based on these findings, 8 mM 2DG was used for all further proliferation studies, as this is the approximate IC_50_ value for both cell lines.

### Effect of 2DG and hypoxia on ATP levels

To measure ATP concentrations cells were exposed to a non-cytotoxic dose (3 mM for 72 h, data not shown) of 2DG either in normoxia or in hypoxia, to ensure that any changes in ATP levels measured were a result of the inhibition of glycolysis and not because of a reduction in cell number (Figure 2B and C). LNCaP cells were more sensitive to hypoxia, with a greater reduction in ATP levels observed both in the presence and in the absence of 2DG in comparison to the MCF-7 cells (−2DG, 9.0 *μ*M±2.1 *vs* 2.9 *μ*M±2.2 SD, *P*<0.05; +2DG, 5.7 *μ*M±1.5 *vs* 3 *μ*M±0.4 SD, *P*<0.05). In contrast, a similar reduction in ATP levels (8.8–12.6 *μ*M) was observed by the addition of 2DG to both cell types in either normoxia or hypoxia. The combination of hypoxia and 2DG reduced ATP levels to 5.1 *μ*M in the MCF-7 cells and to 3.7 *μ*M in the LNCaP cells, a reduction of 75% (^***^P<0.001) and 83% (^***^P<0.001) *vs* untreated, respectively.

### Inhibition of proliferation by 2DG and STX140

**Figure 3:**
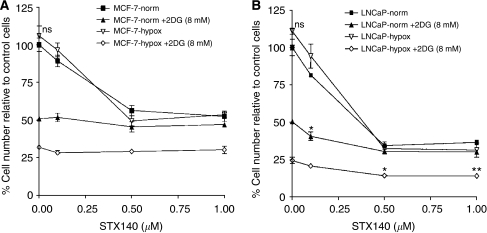
Effects of 2DG and STX140 on the proliferation of MCF-7 (**A**) and LNCaP (**B**) cells. Cells were cultured in 96-well plates, under normoxia or hypoxia, and treated with 2DG (8 mM) and/or STX140 (0.1–1 *μ*M) for 72 h when their effects on proliferation were measured by using a microtitre plate assay. Results are expressed as percent of proliferation of untreated cells in normoxia. In both cell lines 2DG alone was significantly (*P*<0.001) more potent in hypoxia *vs* normoxia. There was no significant benefit of 2DG+STX140 in MCF-7 cells. In LNCaP cells under normoxia, 2DG+0.1 *μ*M STX140 gave significantly greater inhibition than 2DG alone (^*^P<0.05) and 0.1 *μ*M STX140 alone (*P*<0.001). Under hypoxia, 2DG+0.5 *μ*M STX140 and 2DG+1.0 *μ*M STX140 gave significantly greater inhibition than 2DG alone (^*^P<0.05 and ^**^P<0.01) and 0.5 and 1.0 *μ*M STX140 alone (*P*<0.001) (ns=not significant *vs* untreated normoxia).

The growth-inhibitory effects of 2DG and STX140, used alone and in combination, were compared in MCF-7 and LNCaP cells, under both normoxia and hypoxia *in vitro* (Figure 3A and B). The growth inhibition was determined after 72 h. Compared with normoxic untreated controls, STX140 (0.5 *μ*M) inhibited cell proliferation by 45 and 48% in MCF-7 cells (*P*<0.01, Figure 3A) under normoxia and hypoxia, respectively, and by 65% in LNCaP cells (*P*<0.001, Figure 3B) under both normoxia and hypoxia. Under normoxic conditions, in both cell types, 2DG alone (8 mM) inhibited cell proliferation by 50%. Under hypoxic conditions 2DG inhibited cell proliferation by 70 and 75% in MCF-7 and LNCaP cells, respectively, a significant increase in efficacy relative to normoxic conditions (*P*<0.01). Compared with STX140 at 0.5 *μ*M, 2DG (8 mM) alone is significantly more effective at inhibiting tumour cell proliferation in both cell types (MCF-7: *P*<0.01 and LNCaP: *P*<0.05) under hypoxia, but not under normoxia (Figure 3A and B). The addition of STX140 (0.1–0.5 *μ*M) did not further enhance the inhibition of cell proliferation seen with 8 mM 2DG in MCF-7 cells either in normoxia or in hypoxia (Figure 3A). However, improved efficacy was seen when combining the two agents *in vitro* in the LNCaP cells. The combination of 0.1 *μ*M STX140 and 8 mM 2DG was more potent (^*^P<0.05) than either compound alone under normoxia at these concentrations. In contrast, this result was not seen under hypoxic conditions with the same concentrations. However, 8 mM 2DG plus either 0.5 or 1.0 *μ*M STX140 was more potent (^*^P<0.05 and ^**^P<0.01, respectively) than either compound alone under hypoxia at these concentrations; this result was not seen under normoxia with the same concentrations.

### Cell cycle/apoptosis

**Figure 4:**
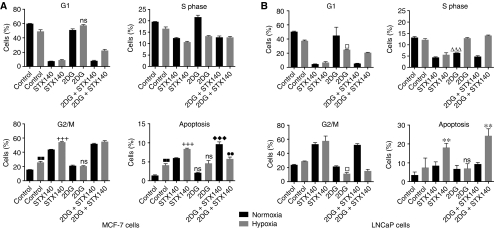
Effects of 2DG, STX140 and hypoxia on cell cycle and apoptosis in MCF-7 (**A**) and LNCaP (**B**) cells. For cell cycle analysis the cells were fixed, treated with RNase, stained with propidium iodide and analysed using a flow cytometer (FACScan, Becton Dickinson, Cowley, UK). For quantification of apoptosis the cells were re-suspended in binding buffer and then stained with fluorescein-conjugated Annexin V antibody and propidium iodide before flow cytometric analysis. Apoptotic cells are defined as cells positive for Annexin V and negative for propidium iodide. All treatments lasted for 48 h (^ΔΔΔ^*P*<0.001 *vs* normoxia control; ^□^*P*<0.05 *vs* normoxia control, hypoxia control and 2DG alone in normoxia; ^▪▪^*P*<0.01 *vs* normoxia control; ^+++^*P*<0.001 *vs* normoxia STX140; ^**^P<0.01 *vs* normoxia and hypoxia control; ^••^*P*<0.01 *vs* STX140 in hypoxia 2DG combined with STX140 under normoxia; ^♦♦♦^*P*<0.001 *vs* normoxia control; and ns=not significant).

To understand the possible mechanisms for STX140/2DG-mediated cell death, both the cell cycle state and mechanism of cell death were assessed by FACS analysis (Figure 4A and B). Earlier studies showed that STX140 induced cell cycle arrest and apoptosis in a range of tumour cell lines (Day *et al*, 2003; Raobaikady *et al*, 2005; Newman *et al*, 2007). In both cell types 2DG alone in normoxia had little effect compared with untreated controls, although a reduction in the S-phase population was seen in LNCaP cells (^ΔΔΔ^*P*<0.001). In LNCaP cells under hypoxia, 2DG alone significantly (^□^*P*<0.05) reduced the number of cells in G1 and G2/M compared with normoxic and hypoxic controls and with 2DG alone in normoxia. Despite this, no significant increase in cells undergoing apoptosis was detected. In MCF-7 cells under hypoxia a small increase was seen in the G2/M and apoptotic cell population compared with normoxia alone (^▪▪^*P*<0.01). Similar data were seen for STX140 in hypoxia compared with STX140 in normoxia in MCF-7 cells (^+++^*P*<0.001). However, 2DG had little effect in MCF-7 cells under hypoxia and the extent of apoptosis measured was the same as seen for hypoxia alone.

In the LNCaP cell line STX140 alone and STX140 with 2DG under hypoxia induced the greatest extent of apoptosis seen in this study; approximately 20% of cells were undergoing apoptosis (^**^P<0.01). In contrast, the greatest extent of apoptosis observed in the MCF-7 line was only 10%, and the combination of hypoxia, 2DG and STX140 was less effective (^••^*P*<0.01) than STX140 alone in hypoxia and 2DG combined with STX140 in normoxia (^♦♦♦^*P*<0.001 *vs* normoxia control).

### Effect of STX140 and 2DG *in vivo*

**Figure 5:**
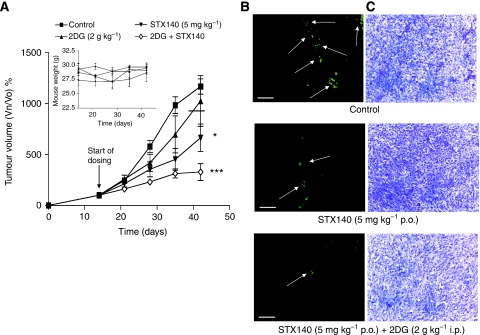
(**A**) Growth of MCF-7 tumours in athymic nude mice. Growth of MCF-7 tumours was inhibited by STX140 (5 mg kg^−1^ p.o.; daily) (^*^P<0.05) and STX140 (5 mg kg^−1^ p.o.; daily) + 2DG (2 g kg^−1^ i.p.; daily) (^***^P<0.001) after 28 days dosing relative to control. There was no effect on mouse weight throughout the study (inset), indicating a potential lack of compound toxicity. Points, mean percentage change in tumour volume; bars, s.e.m. (**B**) von Willebrand's factor staining of tumour sections. Administration of STX140 (5 mg kg^−1^ p.o.; daily) or STX140 (5 mg kg^−1^ p.o.; daily) + 2DG (2 g kg^−1^ i.p.; daily) caused a decrease in blood vessels relative to tumours taken from untreated animals. (**C**) H&E staining of tumour sections. No decrease in overall cell number was observed by H&E staining. Scale bar=100 *μ*M, magnification × 200.

As no previous studies have investigated *in vivo* the combination of a microtubule disruptor and 2DG in breast and prostate cancer, the efficacy of STX140 and 2DG was assessed in the MCF-7 (ER-positive, breast) and LNCaP (AR-positive, prostate) xenograft models. In the breast cancer model (MCF-7) at the end of dosing (day 42), vehicle-treated tumours had increased in size by 1165±75% relative to the tumour starting volumes on day 14. Growth of MCF-7 tumours was significantly inhibited by STX140 (5 mg kg^−1^ p.o.; daily), tumours having increased in size by 664±135% (*P*<0.05). In mice treated with the combination of STX140 (5 mg kg^−1^ p.o.; daily) and 2DG (2 g kg^−1^ i.p.; daily) tumours increased in size only by 328±82% (*P*<0.001). The inset figure reveals that no weight loss occurred, indicating the potential absence of toxicity with these treatments (Figure 5A).

Figure 5B shows representative sections cut from the MCF-7 xenografts stained with an endothelial specific marker, von Willebrand's factor, to assess vessel density. 2-Methoxyoestradiol-3,17-*O*,*O*-*bis*-sulphamate (5 mg kg^−1^ p.o.; daily) and STX140 (5 mg kg^−1^ p.o.; daily) combined with 2DG (2 g kg^−1^ i.p.; daily) caused a reduction in the staining for endothelial cells (indicated by arrows). 2-Deoxy-D-glucose (2 g kg^−1^ i.p.; daily) alone had no effect (data not shown). Conventional H&E staining revealed that the reduction in von Willebrand's staining is not because of a decrease in cell density (Figure 5C).

**Figure 6:**
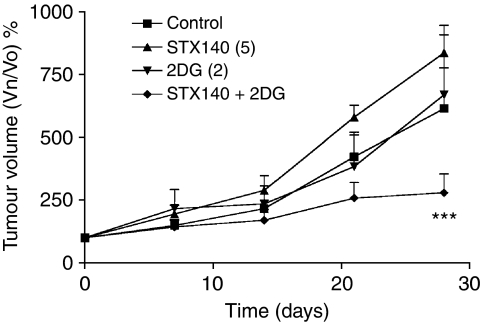
Growth of LNCaP tumours in athymic nude mice. Growth of LNCaP tumours was inhibited by STX140 (5 mg kg^−1^ p.o.; daily) + 2DG (2 g kg^−1^ i.p.; daily) (^***^P<0.001) after 28 days dosing relative to STX140 alone. Points, mean percentage change in tumour volume; bars, s.e.m.

In the prostate cancer model (LNCaP) at the end of dosing (day 28), vehicle-treated tumours had increased in size by 614±162% relative to the tumour starting volumes on day 0. The combination of STX140 (5 mg kg^−1^ p.o.; daily) with 2DG (2 g kg^−1^ i.p.; daily) significantly (*P*<0.001) inhibited tumour growth compared with STX140 alone. No weight loss occurred, indicating the absence of toxicity with these treatments (data not shown) (Figure 6).

## Discussion

Liu *et al* (2001) recently proposed that 2DG combined with a traditional chemotherapeutic agent may offer a new strategy for cancer therapy. They hypothesised that 2DG would target the slowly proliferating cells at the hypoxic centre of the tumour, which are highly dependent on glycolysis, and a chemotherapeutic agent would target the rapidly proliferating cells towards the tumour rim. In our study the combination of STX140 with 2DG was a potent inhibitor of tumour growth, in both breast and prostate cancer xenograft models *in vivo*. The excellent efficacy of this combination dosing supports the original hypothesis of Liu *et al*.

In this study 2DG was combined with STX140, a microtubule disruptor. To date, only one limited study has combined a microtubule disruptor with 2DG. In a NSCLC xenograft model, the combination of 2DG and paclitaxel showed some efficacy (Maschek *et al*, 2004). However, paclitaxel was administered i.p., which was not the optimal route, and no data were shown for the effect of 2DG alone. Furthermore, taxanes may not be the best partner for 2DG, as 2DG induces P-glycoprotein expression, which can expel the taxanes from the cell, leading to a multi-drug-resistant phenotype in the clinic (Ledoux *et al*, 2003; Fojo and Menefee, 2005). In contrast to the taxanes, STX140 is not a substrate for P-glycoprotein and is highly efficacious both *in vitro* and *in vivo* in cells overexpressing P-glycoprotein (Newman *et al*, 2008).

In the MCF-7 breast xenograft model the combination of STX140 and 2DG inhibited tumour growth to the greatest extent; 2DG alone did not have any significant effect on tumour growth. Staining of tumour sections for the endothelial specific marker, von Willebrand's factor, showed a decrease in blood vessel density in response to STX140 alone and the combination of STX140 and 2DG. The decrease in blood vessel density observed agrees with previous studies, which showed a significant reduction in blood vessel density in both the MCF-7 and MDA-MB-231 xenograft models in response to STX140 (Chander *et al*, 2007; Foster *et al*, 2008a). Further work needs to be undertaken to see if the STX140-mediated reduction in blood vessel density increases intratumoral hypoxia, leading to an increased reliance on glycolysis and thus sensitising the cancer cells further to 2DG. In this study, and the studies of Chander *et al* (2007) and Foster *et al* (2008a), daily dosing with STX140 did not cause significant weight loss and there were no gross signs of damage to the normal vasculature, indicating that the efficacious dose of STX140 lacks toxicity.

To test the applicability of this initial *in vivo* finding, STX140 combined with 2DG was tested in an *in vivo* model of prostate cancer, one of the most common cancers in man. In confirmation with the previous data, STX140 and 2DG significantly inhibited LNCaP tumour growth compared with STX140 alone. Monotherapy with 2DG had no significant effect on tumour growth. Unlike the MCF-7 model, no efficacy was seen with STX140 alone, further highlighting the benefit of combining the two agents. Although some studies do report weight loss in response to 2DG (Maschek *et al*, 2004), our data, in both models, concur with those of Gupta *et al* (2005), who reported no weight loss in response to 2DG.

The dose of STX140 used in these combination studies was approximately a quarter of the optimal dose so far identified (Foster *et al*, 2008b), and as such, more work needs to be undertaken to optimise the dosing of this combination treatment. Furthermore, this work indicates that the addition of 2DG to a chemotherapeutic regime may allow for lower doses of chemotherapy to be used, thereby reducing chemotherapy-related toxicity.

To investigate how the combination of 2DG and STX140 may target tumour cells, a series of *in vitro* experiments were undertaken; in these studies a hypoxic incubator was used to try and model the inner core of the tumour. The MCF-7 and LNCaP cell lines were equally sensitive to 2DG alone *in vitro*, with approximate IC_50_ values of 8 mM being obtained for both cell lines in normoxia. This value is in agreement with other studies that have shown IC_50_ values in the range of 4–12 mM in a variety of cell lines (Kaplan *et al*, 1990; Aft *et al*, 2002; Lampidis *et al*, 2006). The relatively high IC_50_ values for the inhibition of proliferation reflect the fact that 2DG has to compete with glucose in the culture medium for uptake into the cells. When the cells were grown under hypoxic conditions their sensitivity to 2DG significantly increased. These data suggest that under hypoxia the cells become more reliant on glycolysis and are therefore more sensitive to 2DG. This is supported by the significant reduction in cellular ATP levels seen in response to hypoxia alone, and the further reduction in ATP levels observed when hypoxia is combined with 2DG. Even in normoxia both these cell lines appear to be reliant on glycolysis to some extent, as 2DG alone in normoxia reduces ATP production by over 50% compared with normoxic controls. The combination of STX140 with 2DG only enhanced the inhibition of cell proliferation seen with 2DG alone, in the LNCaP cell line, at low doses of STX140 in normoxia and only at higher doses of STX140 in hypoxia. This result was supported by the significant increase in apoptosis seen with STX140 and 2DG under hypoxia compared with other treatments in the LNCaP cells, but not in the MCF-7 cells. These *in vitro* data further support the hypothesis of Liu *et al*. (2001), as the chemotherapeutic agent, STX140, is equally efficacious in normoxia and hypoxia but 2DG is more efficacious in the hypoxic environment, which is hypothesised to reflect the centre of the tumour.

This study for the first time shows the benefit of combining 2DG with an antiproliferative compound *in vivo* in the two most common cancers. This work supports the continued investigation into the use of 2DG combined with chemotherapeutic drugs for the treatment of cancer.
